# T2-mapping in volunteers: influence of sequence, spatial orientation and interindividual variability

**DOI:** 10.1186/1532-429X-13-S1-P61

**Published:** 2011-02-02

**Authors:** Ralf Wassmuth, Jeanette Schulz-Menger

**Affiliations:** 1 Charite, University Berlin, Berlin, Germany

## Background

CMR T2-mapping is a promising tool for characterizing myocardial edema. We applied T2-mapping in volunteers to compare two mapping sequences and to assess feasibility, reproducibility and spatial homogeneity.

## Methods

We scanned 26 volunteers (10 female, 20-70 years, mean 32±13 years, median 28 years, BMI 23±3 kg/m2) with a normal ECG, no cardiac disease and no symptoms of inflammation.

Using a 1.5 T scanner and a dedicated 12-element cardiac coil we applied a FLASH-based and SSFP-based mapping sequence in midventricular short axis (SAX) and four-chamber-view (4CV). The map was based on three images with an echo time of 0, 24 and 55 ms. Spatial resolution was 2.1 mm/pixel. Scan time was 12 heart beats.

In Osirix 3.3.2. we manually drew 6 segments in SAX and 4CV, each. Additionally, we drew a global region of interest (ROI) covering the whole LV myocardium in SAX and 4CV. The same investigator analyzed 10 data sets twice. 5 volunteers were scanned twice on separate days. Results were compared with a paired student t-test.

## Results

After excluding one subject due to obvious pathologies, 25 datasets with sufficient image quality were evaluated. Table 1 gives mean and range for global ROI.

**Table 1:** 

	FLASH	SSFP	
SAX	52±8; 42-60	57±10; 46-69	p< 0.001
4CV	58±10; 47-74	61±14; 51-80	p<0.005
	p<0.001	p<0.001	

FLASH and SSFP correlated better in 4CV than in SAX (correlation coefficient 0.92 vs. 0.80; p< 0.04). T2-values did not correlate with heart rate (p=0.3).

With both sequences anteroseptal segments had higher T2-values than inferior and inferolateral segments in SAX (for FLASH 58±6 vs. 48±4; p< 0.001). In 4CV the basal septum had higher T2-values than the anterolateral segment with FLASH (62±7 vs. 54±8 ms; p<0.001), but not with SSFP (58±6 vs. 60±11 ms; p=0.3). Mean absolute difference between a single segment and a global measurement was 4±1 ms and 3±1 ms for FLASH and SSFP in SAX and 5±2 ms for FLASH and SSFP in 4CV (p=0.3).

Mean difference for repeated analysis was 1.6±1.9 ms (correlation coefficient 0.9) and 2.2±2.2 ms (correlation coefficient 0.7) for repeated scans.

## Conclusion

T2-mapping is feasible with low intraobserver variability and does not depend on heart rate. SSFP-based T2-mapping resulted in slightly higher values than FLASH. Mapping in 4CV resulted in higher T2-values than in SAX. We could detect small spatial differences across the heart. However, these intraindividual spatial variations were smaller than considerable interindividual variability.

